# High‐Contrast Imaging of α‐Synuclein Pathologies in Living Patients with Multiple System Atrophy

**DOI:** 10.1002/mds.29186

**Published:** 2022-08-30

**Authors:** Kiwamu Matsuoka, Maiko Ono, Yuhei Takado, Kosei Hirata, Hironobu Endo, Toshiyuki Ohfusa, Taichi Kojima, Takeshi Yamamoto, Tomohiro Onishi, Asumi Orihara, Kenji Tagai, Keisuke Takahata, Chie Seki, Hitoshi Shinotoh, Kazunori Kawamura, Hiroshi Shimizu, Hitoshi Shimada, Akiyoshi Kakita, Ming‐Rong Zhang, Tetsuya Suhara, Makoto Higuchi

**Affiliations:** ^1^ Department of Functional Brain Imaging, Institute for Quantum Medical Science, Quantum Life and Medical Science Directorate National Institutes for Quantum Science and Technology Chiba‐shi Japan; ^2^ Department of Psychiatry Nara Medical University Kashihara‐shi Japan; ^3^ Neurology Tsukuba Research Department, Discovery, Medicine Creation, Eisai Co., Ltd. Tsukuba‐shi Japan; ^4^ Translational Research Laboratories, Ono Pharmaceutical Co. Ltd. Shimamoto‐cho, Mishima‐gun Japan; ^5^ Neuroscience Drug Discovery Unit, Research Takeda Pharmaceutical Company Limited Fujisawa‐shi Japan; ^6^ Neurology Clinic Chiba Chiba‐shi Japan; ^7^ Department of Advanced Nuclear Medicine Sciences, Institute for Quantum Medical Science, Quantum Life and Medical Science Directorate National Institutes for Quantum Science and Technology Chiba‐shi Japan; ^8^ Department of Pathology, Brain Research Institute Niigata University Niigata‐shi Japan; ^9^ Center for Integrated Human Brain Science, Brain Research Institute Niigata University Niigata‐shi Japan

**Keywords:** α‐synuclein, multiple system atrophy, PET, neuroimaging

Multiple system atrophy (MSA) is a neurodegenerative disorder characterized by glial cytoplasmic inclusions (GCIs)^1^ composed of α‐synuclein aggregates as neuropathological hallmarks.^2^ In vivo visualization of α‐synuclein pathologies potentially offers diagnostic assessments of MSA, but has been challenging because of the lack of sensitive imaging agents.

We developed a new small‐molecule ligand for α‐synuclein fibrils, ^18^F‐SPAL‐T‐06 (PCT/JP2021/030899), as a candidate positron emission tomography (PET) probe for α‐synucleinopathies. Non‐clinical characterization of this compound was performed as described in our assessments of other α‐synuclein imaging probes.^3^ Here, we report our first‐in‐human PET study for patients with two MSA subtypes.

We enrolled three patients with the clinical diagnosis of probable MSA according to the second consensus statement on the diagnosis of MSA,^4^ along with a 72‐year‐old healthy control (HC). All subjects were judged as amyloid‐negative by visual assessment of ^11^C‐Pittsburgh compound‐B‐PET images. The patients consisted of two cases with MSA with predominant parkinsonism (MSA‐P) and one case with MSA with predominant cerebellar ataxia (MSA‐C). Detailed clinical and demographic information on these individuals is provided in Supporting Data. This study was approved by the certified review board (jRCTs031210180).

We performed PET scans with ^18^F‐SPAL‐T‐06 for all subjects and used PET data at 100–120 min after the radioligand injection to estimate the radioligand retention as standardized uptake value ratio to the cerebellar cortex, which was conceived to bear no marked α‐synuclein burden. Notably, we observed enhanced ^18^F‐SPAL‐T‐06 retentions in the putamen, pons, and cerebellar white matter and peduncles of MSA‐P and MSA‐C cases in sharp contrast to minimal radiosignals in the corresponding areas of the HC (Fig. 1A). The topology of the increased radioligand binding accordingly agreed with the predominant distributions of GCIs in patients with these MSA subcategories.^5^

**FIG 1:**
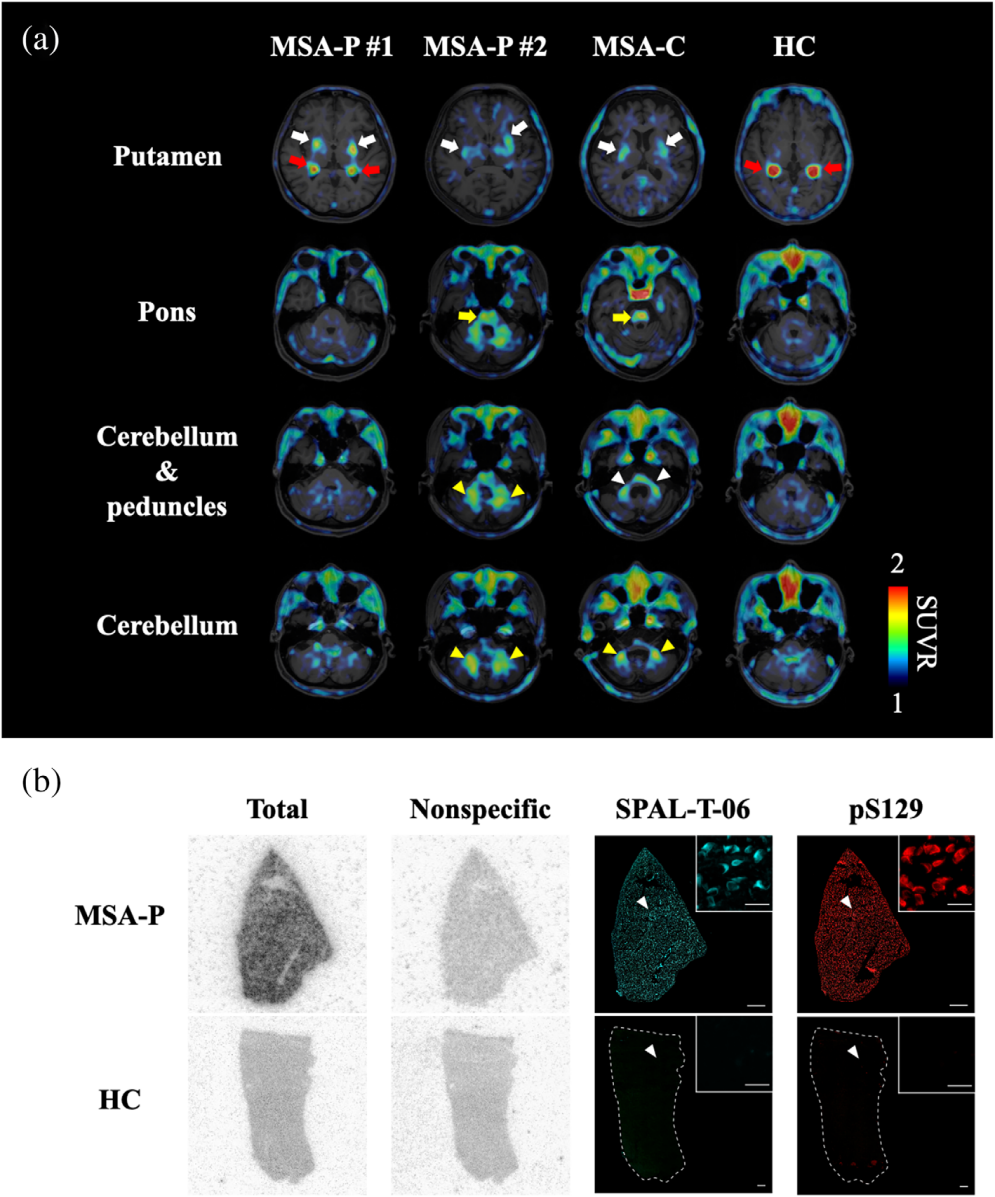
In vivo and in vitro binding of SPAL‐T‐06 to α‐synuclein pathologies in MSA patients. (**A**) Axial ^18^F‐SPAL‐T‐06‐PET images of the brains of the MSA patients and HC. Data are displayed as parametric maps for radioligand SUVRs at 100–120 min after the radioligand injection (dose, 176.9 ± 4.9 MBq; molar activity, 237.5 ± 53.9 GBq/μmol). All MSA patients showed augmented ^18^F‐SPAL‐T‐06 retentions in the putamen (white arrows), whereas the MSA‐P case 2 and MSA‐C case presented elevated ^18^F‐SPAL‐T‐06 accumulations in the pons (yellow arrows) and cerebellar white matter (yellow arrowheads). Moreover, the radioligand binding was enhanced in the cerebellar peduncles of the MSA‐C case (white arrowhead). Red arrows denote nonspecific radioactivity accumulations in the choroid plexus. (**B**) Autoradiographic labeling of putamen sections derived from MSA‐P (upper row) and HC (lower row) cases with 1 nM of ^18^F‐SPAL‐T‐06 in the absence and presence of 10 μM of non‐radiolabeled SPAL‐T‐06 displaying the total (1st column from the left) and non‐specific (2nd column) radioligand binding, respectively. These sections were then used for fluorescence staining with non‐radiolabeled SPAL‐T‐06 (30 μM) (3rd column) and pS129 (4th column), revealing abundant GCIs in the putamen of the MSA‐P case. Arrowheads indicate the location of the area magnified in the insets. Scale bars, 1 mm (low‐power fields); 20 μm (insets). HC, healthy control; MSA, multiple system atrophy; MSA‐C, multiple system atrophy with predominant cerebellar ataxia; MSA‐P, multiple system atrophy with predominant parkinsonism; SUVR, standardized uptake value ratio. [Color figure can be viewed at wileyonlinelibrary.com]

Before these clinical PET assessments, in vitro binding characteristics of ^18^F‐SPAL‐T‐06 were examined using postmortem MSA‐P and HC brain tissues from the brain bank at Niigata University. Autoradiography demonstrated homologously displaceable binding of ^18^F‐SPAL‐T‐06 in the putamen of the MSA‐P case in line with dual‐labeling of numerous GCIs in the same section with SPAL‐T‐06 fluorescence and an anti‐phosphorylated α‐synuclein antibody, pS129 (Fig. 1B). By contrast, there was no noticeable autoradiographic and fluorescent labeling in the HC putamen. Moreover, we quantified the affinity of ^18^F‐SPAL‐T‐06 in homogenates of the MSA‐P putamen as described elsewhere,^3^ and calculated dissociation constant was 2.49 nM (Supplementary Fig. S1), indicating high reactivity of this radioligand with MSA‐type α‐synuclein assemblies. We also observed minimal displacement of the ^18^F‐SPAL‐T‐06 binding with inhibitors of monoamine oxidases A and B in the MSA‐P putamen homogenates, which is indicative of negligible cross‐reactivity of ^18^F‐SPAL‐T‐06 with these off‐target components (Supplementary Fig. S1).

The present study provides the first in vivo demonstration of PET imaging of α‐synuclein pathologies in MSA‐P and MSA‐C patients with high contrast, allowing visual read of images in each individual for a diagnostic purpose. Encouraged by the current findings, PET assays of the ^18^F‐SPAL‐T‐06 binding in allied α‐synucleinopathies exemplified by idiopathic Parkinson's disease and dementia with Lewy bodies are underway.

## Financial Disclosures

K. M., K. Tagai, and K. K. were employed by QST and were supported by JSPS KAKENHI. M. O. and Y. T. were employed by QST and supported by JSPS KAKENHI and AMED. K. H. was supported by JPSP KAKENHI. H. E. was employed by QST, was supported by JSPS KAKENHI, and was a consultant on image analysis for APRINOIA Therapeutics. T. Ohfusa was employed by Eisai Co., Ltd. T. K. was employed by Ono Pharmaceutical Co. Ltd. T. Y. and T. Onishi were employed by Takeda Pharmaceutical Company Limited. A. O. and C.S. were employed by QST. K. Takahata was employed by QST and was supported by JSPS KAKENHI and MHLW JPMH. H. Shinotoh was employed by Neurology Clinic Chiba. H. Shimizu and A.K. were employed by Niigata University and were supported by JSPS KAKENHI. H. Shimada was employed by Niigata University, was supported by JSPS KAKENHI, and held patents (JP 5422782/EP 12 884 742.3/CA2894994/HK1208672/ZL201710407246.4). M. R. Z. was employed by QST, was supported by JSPS KAKENHI and AMED, and held patents (JP 5422782/EP 12 884 742.3/CA2894994/HK1208672/ZL201710407246.4). T. S. was employed by QST, was supported by AMED, and held patents (JP 5422782/EP 12 884 742.3/CA2894994/HK1208672/ZL201710407246.4). M. H. was employed by QST, was supported by JSPS KAKENHI, AMED, and JST CREST, and held patents (JP 5422782/EP 12 884 742.3/CA2894994/HK1208672/ZL201710407246.4).

## Author Roles

(1) Research Project: A. Conception, B. Organization, C. Execution; (2) Data Analysis: A. Design, B. Execution, C. Review and Critique; (3) Manuscript: A. Writing of the First Draft, B. Review and Critique.

K.M.: 1A, 1B, 1C, 2A, 2B, 2C, 3A, 3B.

M.O.: 1A, 1B, 1C, 2A, 2B, 2C, 3A, 3B.

Y.T.: 1A, 1B, 1C, 2A, 2C, 3A, 3B.

K.H.: 1C, 3A, 3B.

H.E.: 1C, 3A, 3B.

T.Ohfusa: 2A, 2B, 2C, 3B.

T.K.: 2A, 2B, 2C, 3B.

T.Y.: 2A, 2B, 2C, 3B.

T.Onishi: 2A, 2B, 2C, 3B.

A.O.: 1C, 3B.

K.Tagai: 1C, 3B.

K.Takahata: 1C, 3B.

C.S.: 1C, 2A, 2B, 2C, 3B.

H.Shinotoh: 1C, 3B.

K.K.: 1C, 2A, 2B, 2C, 3B.

H.Shimizu: 1C, 2A, 3B.

H.Shimada: 2C, 3B.

A.K.: 1C, 2A, 3B.

M.R. Z.: 1C, 2A, 2C, 3B.

T.S.: 2C, 3B.

M.H.: 1A, 1B, 1C, 2A, 2C, 3A, 3B.

## Ethics Statement

The present study was approved by the National Institutes for Quantum Science and Technology Certified Review Board. We obtained written informed consent from all the subjects.

## Supporting information


**Figure S1** Total binding of 1 nM of ^18^F‐SPAL‐T‐06 in homogenates of the MSA‐P putamen under homologous and heterologous blockade conditions. The radioligand binding was homologously blocked by non‐radiolabeled SPAL‐T‐06 in a concentration‐dependent fashion (blue symbols) with a dissociation constant of 2.49 nM. By contrast, the total binding was inhibited by neither clorgiline, a monoamine oxidase‐A inhibitor (red symbols), nor selegiline, a monoamine oxidase‐B inhibitor (green symbols), at varying concentrations. Data are mean values ± SD and are expressed as % of the averaged total binding without blockades.

## Data Availability

Data are available upon reasonable request. Anonymized raw data supporting the findings of the present study may be shared upon request to the corresponding author.
