# Supplementary material for: High‐Contrast Imaging of α‐Synuclein Pathologies in Living Patients with Multiple System Atrophy
Source: Mov Disord. 2022 Aug 30;37(10):2159–61. doi: 10.1002/mds.29186 (PMC9804399; doi:10.1002/mds.29186)

**Supplemental Materials**

**Demographic and clinical information of the MSA cases**

*MSA-P #1*

The first case diagnosed as having MSA-P was a 56-year-old woman with a 4-year history of progressive parkinsonism, including left-predominant limb rigidity, bradykinesia, postural instability, and orthostatic hypotension.

*MSA-P #2*

The second MSA-P was a 61-year-old woman with a 3-year history of progressive parkinsonism, including right limb rigidity and bradykinesia, orthostatic hypotension, and dysuria.

*MSA-C*

The case diagnosed as having MSA-C was a 70-year-old woman with a 4-year history of progressive cerebellar gait ataxia and left-predominant limbs ataxia. The neurologic examination revealed slurred speech, orthostatic hypotension, dysuria, and mild limb rigidity.

**Supplementary Figure 1**

Total binding of 1nM of ^18^F-SPAL-T-06 in homogenates of the MSA-P putamen under homologous and heterologous blockade conditions. The radioligand binding was homologously blocked by non-radiolabeled SPAL-T-06 in a concentration-dependent fashion (blue symbols) with a dissociation constant of 2.49 nM. By contrast, the total binding was inhibited by neither clorgiline, a monoamine oxidase-A inhibitor (red symbols), nor selegiline, a monoamine oxidase-B inhibitor (green symbols) at varying concentrations. Data are mean values ± SD and are expressed as % of the averaged total binding without blockades.


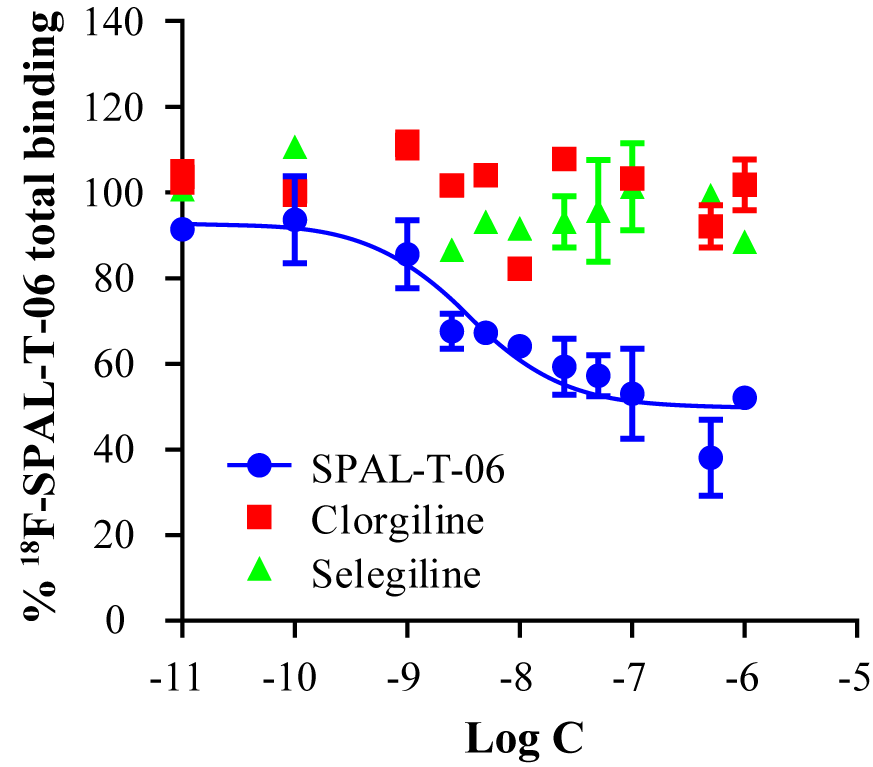

Supplement: Supplementary file 1 — Figure S1 Total binding of 1 nM of 18F‐SPAL‐T‐06 in homogenates of the MSA‐P putamen under homologous and heterologous blockade conditions. The radioligand binding was homologously blocked by non‐radiolabeled SPAL‐T‐06 in a concentration‐dependent fashion (blue symbols) with a dissociation constant of 2.49 nM. By contrast, the total binding was inhibited by neither clorgiline, a monoamine oxidase‐A inhibitor (red symbols), nor selegiline, a monoamine oxidase‐B inhibitor (green symbols), at varying concentrations. Data are mean values ± SD and are expressed as % of the averaged total binding without blockades. [file MDS-37-2159-s001.docx]
